# A statistical assessment of differences and equivalences between genetically modified and reference plant varieties

**DOI:** 10.1186/1472-6750-11-15

**Published:** 2011-02-16

**Authors:** Hilko van der Voet, Joe N Perry, Billy Amzal, Claudia Paoletti

**Affiliations:** 1 Wageningen University and Research centre, Biometris, P.O. Box 100, NL-6700 AC Wageningen, Netherlands; 2 Oaklands Barn, Lug's Lane, Broome, Norfolk NR35 2HT, UK; 3 European Food Safety Authority, Largo N. Palli 5/A, 43121 Parma, Italy

## Abstract

**Background:**

Safety assessment of genetically modified organisms is currently often performed by comparative evaluation. However, natural variation of plant characteristics between commercial varieties is usually not considered explicitly in the statistical computations underlying the assessment.

**Results:**

Statistical methods are described for the assessment of the difference between a genetically modified (GM) plant variety and a conventional non-GM counterpart, and for the assessment of the equivalence between the GM variety and a group of reference plant varieties which have a history of safe use. It is proposed to present the results of both difference and equivalence testing for all relevant plant characteristics simultaneously in one or a few graphs, as an aid for further interpretation in safety assessment. A procedure is suggested to derive equivalence limits from the observed results for the reference plant varieties using a specific implementation of the linear mixed model. Three different equivalence tests are defined to classify any result in one of four equivalence classes. The performance of the proposed methods is investigated by a simulation study, and the methods are illustrated on compositional data from a field study on maize grain.

**Conclusions:**

A clear distinction of practical relevance is shown between difference and equivalence testing. The proposed tests are shown to have appropriate performance characteristics by simulation, and the proposed simultaneous graphical representation of results was found to be helpful for the interpretation of results from a practical field trial data set.

## Background

Biotechnology has developed to allow the production of genetically modified organisms carrying specific characteristics of interest. For example, plants can be made tolerant to a herbicide, thus facilitating traditional chemical weed control, or can be induced to produce an insecticidal protein, thus reducing the need for external chemical treatment. Because of its novelty, concerns exist regarding the safety of genetic modifications (e.g. [1]).

In Europe, genetically modified organisms (GMOs) and derived products are allowed on the market after passing an approval system in which the safety for humans, animals and the environment is assessed. This safety assessment is performed by the GMO Panel of the European Food Safety Authority (EFSA), that has issued guidance for applicants who seek to bring GMOs onto the European market [2]. These guidelines advocate a risk assessment strategy known as the comparative approach [3,4]. Comparative risk assessment is based on the idea that typically there are organisms that are very similar to the GMO, which have a history of safe use as foods. Such organisms can be used as comparators for the GMO, and the purpose of the comparative analysis is to identify similarities and differences between the GM crop-derived food/feed and its non-GM counterparts. In the first step agronomic and morphological characteristics of plants are considered as well as their chemical composition. The general idea is that a comparative risk assessment can establish equivalence between the GMO and its non-GM counterpart for characteristics other than the intended effects of the genetic modification. Equivalence in this context is defined as the absence of differences other than those due to natural biological variation. However, little guidance is available how to perform equivalence testing for GMOs in practice. Although the EFSA Guidance Document [2] discusses general principles for risk assessment and recommends the use of appropriate statistical tools, detailed protocols for the design of experiments and statistical analysis are not provided.

In practice, applicants have been using widely differing protocols to carry out field trials and very different models for the statistical analysis (e.g. [5-10]). Oberdoerfer et al. [5] applied equivalence tests using fixed but arbitrary equivalence limits of ± 20%. In a later paper by Hothorn and Oberdoerfer [6] this fixed value was described as rigid and not reflecting the difference in variability between components, and component-specific safety ranges were proposed to be proportional to the variance of the concurrent control in the same field trials, which method ignores the amount of background variation found between commercial varieties. Hammond et al. [7] and Park et al. [8] only applied difference tests, which ignores the question which difference would be relevant biologically. Special pleading for significant results was needed in the first of these publications to arrive at their conclusion that the GMO was as safe and nutritious as reference varieties. McNaughton et al. [9] and Herman et al. [10] adjusted p values of difference tests using the False Discovery Rate method which is a method developed for multiple difference tests. However, this method is simply not appropriate in the context of equivalence testing because it is only concerned with false discoveries and not with false non-discoveries. In any case, whatever the advantages and disadvantages of the methods, the application of different statistical approaches and models may lead to different conclusions regarding the risk assessment of GMOs and derived foods/feeds. Therefore a working group of the EFSA GMO Panel investigated whether more detailed guidance could be provided to applicants regarding the use of appropriate statistical models for the analysis of the data from field trials for compositional, agronomic and phenotypic studies and animal feeding trials, and regarding the design of field trials. This paper is based on the report of the working group [11], and is restricted to the statistical analysis of data on the chemical composition of plants obtained in field trials. We will not further discuss the experimental design of such field trials (see [11]). Whereas typically many plant characteristics are analysed in such field trials, the scope of the statistical tests in this paper is restricted to the analysis of single characteristics. However, a simultaneous display of test results for multiple plant characteristics is advocated.

Equivalence testing is commonly used in biomedical and pharmaceutical statistics [12,13]. For example, pharmacokinetic parameters of alternative drug formulations have to be shown to be within a factor 1.25 of the value for the reference drug. In words, the null hypothesis of the equivalence test is (for the symmetric case) "there is a difference between the GMO and its reference of a certain minimum size" against the alternative hypothesis: "there is no or only a small difference between the GMO and its reference". In this testing procedure we need a significant result (rejection of the null hypothesis) in order to conclude that the GMO and the reference are equivalent. Thus there is a limited Error I probability (α) that equivalence is concluded whereas a difference larger than the limit value exists in reality.

Specific questions exist for equivalence testing of GMOs. First, how can equivalence limits for GMO mean characteristics relative to reference mean characteristics be defined given that no generally agreed equivalence factor (such as 1.25) exists? Secondly, how should equivalence limits be estimated given that appropriate data from field trials will describe typical biological variation rather than the maximum allowable variation? Thirdly, can the equivalence testing procedure be adapted to the typical large biological variation usually found in field studies?

Equivalence testing requires the determination of equivalence limits to enable its implementation. For each chosen characteristic, or for groups of them, equivalence limits for the true difference are effectively the limiting values for an acceptable difference. In this paper we will determine equivalence limits as relative deviations from the overall mean of the reference varieties. In current practice, equivalence limits have almost never been established satisfactorily before the experiment. Therefore, we suggest that commercial reference varieties are included in field experiments, to allow a direct comparison with the GMO. As will be explained, this can be seen as a test of the difference between GMO and the population of commercial references. An alternative view is that the reference varieties in the experiment allow the estimation of equivalence limits, which are subsequently used for assessing the equivalence of the GMO. Inclusion of both the conventional counterpart and reference varieties in field or animal experiments for GMO safety testing is not new, and papers on such experiments have been published (e.g. [14-16]). However, as far as we know data from such studies have not been used before for setting limits for equivalence testing in the manner proposed here.

## Results

### Equivalence testing for GMOs

We developed a statistical methodology to assist in the comparative risk assessment of GM crops. The novelty of the method is the simultaneous assessment of both differences and similarities (equivalences). To detect unintended effects, it is optimal to study the differences of the GMO with its non-GM counterpart. However, to assess similarities and equivalence, a characterization of natural biological variability is needed. We propose that the GMO can be viewed relative to the background variation shown by common varieties (e.g. commercial varieties) used as references. For this dual purpose of difference and equivalence testing we propose the use of field trials using not only the GM crop and its non-GM counterpart, but also a range of reference varieties. Designs using a wide range of varieties have been used previously, showing that this is a feasible approach in principle (e.g. [14-17]).

When testing for differences (proof of difference approach) the null hypothesis and alternative hypothesis are:

$$\begin{array}{l} H_{0}:\;\Delta_{GC}=0\\ H_{1}:\;\Delta_{GC}\neq 0 \end{array}$$

where Δ_GC_ is the true difference on an appropriate scale between the GMO and the conventional counterpart. Student's t-test is a common tool for simple comparisons, but for the statistical analysis of data from more complicated designs linear mixed models are appropriate, as are also used in this paper (see Methods section). For testing statistical significance of differences an alternative procedure is to construct a confidence interval for the difference, and observe whether this includes the value zero. This method is preferred because it gives the bonus of a quantification of the estimated difference and its uncertainty.

A statistically significant test result identifies a difference, whether it is biologically relevant or not. Moreover, whereas for tests with confidence level 1-α there is a limited Error I probability (α) that a significant result is obtained (i.e. a difference is found) whereas no difference exists in reality, these tests do not restrict the Error II probability (β) of finding no significance whereas in reality there is a difference. So the absence of a significant difference is not a proof for equivalence of the GMO and the counterpart, or ''absence of evidence is not evidence of absence'' [18,19]. This motivates supplementing the difference test with an equivalence test.

For equivalence testing the state of non-equivalence needs to be put as a null hypothesis, thus assigning the restricted Error I probability α of the test to the event of falsely declaring equivalence (rejecting the null hypothesis of non-equivalence). This requires the use of equivalence limits as maximum acceptable deviations because the simple inequality Δ≠0 does not qualify as a testable hypothesis. Thus, when testing for equivalence of a GMO and a reference the null and alternative hypotheses become:

$$\begin{array}{l} H_{0}:\;\;\;|\Delta_{GR}|\;\;\geq EL\;\\ H_{1}:\;|\Delta_{GR}|\;<EL \end{array}$$

where Δ*_GR _*is the true difference between the GMO and the reference, and where *EL *is the equivalence limit for this difference. Note that the hypotheses above assume symmetrical lower and upper equivalence limits (*EL_L _*= -*EL *and *EL_U _*= -*EL*), but this can be easily generalised if needed. Using this set of hypotheses it can be seen that the observed difference between GMO and reference mean should be small to reject the null hypothesis (and therefore accept the alternative statement of equivalence). Large differences will not lead to significant test results (and therefore the statement of non-equivalence cannot be rejected in those cases).

With prior specified equivalence limits, both the difference test and the set of equivalence tests can be implemented using a single confidence interval. This is in the spirit of the two one-sided tests (TOST) approach of Schuirmann [20]. Both implied null hypotheses of non-equivalence (*H*_0_: Δ*_GR _*≤ -*EL *and *H*_0_: Δ*_GR _*≥ *EL*) are rejected if the confidence interval lies entirely between the equivalence limits. In equivalence studies the choice of a 90% confidence interval is customary [12,13] as it corresponds with the customary 95% level for statistical testing of equivalence. However, it should be stressed that preference for specific levels of confidence is not a statistical decision, but one to be made by risk managers. For simplicity of the approach we nevertheless propose to calculate by default two-sided 90% confidence intervals rather than calculating confidence intervals separately with different confidence levels for the difference and the equivalence tests. This proposal implies that each (two-sided) difference test will have a 90% confidence level, and each equivalence test a 95% confidence level.

The statistical procedure needed for GMO equivalence testing is however more complicated than this. A single test would be sufficient with fixed equivalence limits, using e.g. a generally agreed equivalence factor (such as 1.25). Lacking this we estimate equivalence limits from field studies with concurrent reference varieties, which are typically the same studies in which also the GMO and its non-GM counterpart are tested. We therefore have a two-step procedure, at least in principle. The first step is the setting of equivalence limits (step L), the second step is their use for assessing equivalence (step E).

As practical limits on background variation we consider appropriate percentiles (e.g. 2.5 and 97.5) of the distribution of reference variety characteristics as the true equivalence limits. Being based on limited data, the *estimated *equivalence limits in step L are always uncertain. In principle, equivalence limits could be calculated in one of the following three ways: 1) as point estimates of the true equivalence limits; 2) as 'inner' confidence limits (by setting them as the lower confidence limit on the upper equivalence limit and the upper confidence limit on the lower equivalence limit); or 3) as 'outer' confidence limits (the upper confidence limit on the upper equivalence limit and the lower confidence limit on the lower equivalence limit). In this paper the third option is chosen, because typical variation between reference varieties is smaller than maximum allowable variation, which would ideally underlie the setting of equivalence limits. Consequently equivalence limits based on the typical variation between reference varieties in the field trial are not true safety limits but only specifications of limits on natural background variation. Therefore their uncertainty can be allowed to be included in the width of the equivalence interval as in the chosen third option. The EFSA GMO Panel considered that specifying minimum requirements for the experimental design [11] was enough to limit the inevitable estimation errors to reasonable levels.

The second step of the equivalence testing procedure (step E) consists of comparing the GMO mean to the equivalence limits obtained in step L. Again there are three options for testing: (E1) direct comparison of the point estimate for the GMO to the equivalence limits; (E2) a true test of equivalence (test the null hypothesis that the GMO mean is outside the equivalence limits against the alternative that it is inside them); and (E3) a true test of non-equivalence (test the null hypothesis that the GMO mean is inside the equivalence limits against the alternative that it is outside them). Borrowing some terminology from quality inspection theory (e.g. [21]), test E2 controls the 'consumer's risk' because it has a limited probability of accepting non-equivalent varieties, and test E3 controls the 'producer's risk' because it has a limited probability of rejecting acceptable varieties. Test E1 will find 'equivalence' more often than test E2 but less often than test E3, for which reason we will refer to test E1 as a 'shared risk' test. 'Shared' here means that a borderline variety has a 50% probability to be classified as either equivalent or non-equivalent using this test procedure (as is confirmed by simulation, see Table 1). We propose to classify the results of test E1 as 'equivalence more likely than not' or 'non-equivalence more likely than not'.

In traditional equivalence testing [12,13] E2-type tests are being used. However, these may have a low power in case of a large residual variation, which is typical for agricultural field studies. Therefore, addressing our third question (how to counter the problem of high variability), it is proposed here not only to rely on test E2, but to apply all three tests. This provides a richer view on equivalence than obtained by using only one test. Therefore the final outcome of the equivalence assessment is not just binary, but it is one of four equivalence categories, for which we propose the following labels: (i) equivalence; (ii) equivalence more likely than not; (iii) non-equivalence more likely than not; and (iv) non-equivalence.

The outcome of test E2 discriminates category (i) from (ii)+(iii)+(iv). Similarly, the outcome of test E3 discriminates category (iv) from (i)+(ii)+(iii). The outcome of test E1 discriminates (i)+(ii) from (iii)+(iv). In the Method section exact calculations will be defined, and it will be shown that the proposed test E1 applied to estimated equivalence limits is actually just a test of difference, with the null hypothesis that GMO and reference means are equal, but allowing for variability between genotypes (which is the crucial difference with the traditional difference test). On the other hand, tests E2 and E3 are truly two-step procedures, where the null hypothesis value of the test is only established after step L. Consequently, statistical properties of such tests can only be defined conditionally on the outcome of step L.

**Figure 1:**
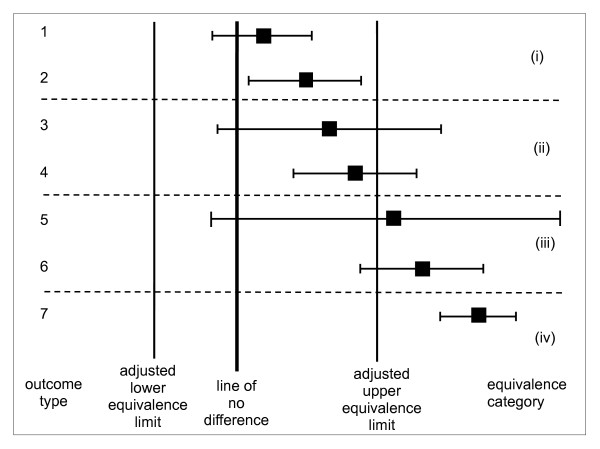
**Simplified version of a graph for comparative assessment showing the seven outcome types possible for each characteristic analysed**. After adjustment of the equivalence limits, a single confidence limit (for the difference) serves visually for assessing the outcome of both difference and equivalence tests. Here, only the upper adjusted equivalence limit is considered. Shown are: the mean of the GMO compared to the mean of the counterpart on an appropriate scale (square), its confidence interval (bar), a thick vertical line indicating zero difference (for proof of difference), and thinner vertical lines indicating adjusted equivalence limits on the same scale (for proof of equivalence). For outcome types 1, 3 and 5 the null hypothesis of no difference cannot be rejected: for outcomes 2, 4, 6 and 7 the GMO is different from its counterpart. Regarding interpretation of equivalence, four categories (i) - (iv) are identified: in categories (i) and (iv) there is a significant equivalence and non-equivalence, respectively, in categories (ii) and (iii) equivalence and non-equivalence, respectively, are more likely than not.

For the interpretation of results we recommend a graphical display, similar to those suggested by others [22,23]. However, certain adjustments are needed to account for the fact that equivalence limits are estimated values, and these are described in detail in the Methods section. Figure 1 presents a schematic simplified example of the display, showing the possible outcomes for a single characteristic. For any given characteristic there are then fundamentally seven possible types of outcome. Among these seven types there are four where the mean value of the GMO lies between the adjusted equivalence limits (types 1-4), and three where it lies outside the equivalence limits (types 5-7). It is assumed here that the line of no difference is in between the adjusted equivalence limits. If not, then the selected conventional counterpart is itself non-equivalent to the reference varieties and a separate, non-statistical discussion should consider the place and relative importance of difference and equivalence testing in the risk assessment.

The results of the four tests are easily derived from the graphical presentation as in Figure 1. The test of difference will give a significant result if the confidence interval bar does not cross the line labelled "no difference". Therefore in outcome types 2, 4, 6 and 7 there is a significant difference between the GMO and the counterpart. The test of equivalence consists of three subtests as explained before. First, when the point estimate of the GMO vs. counterpart difference falls within the adjusted equivalence limits then the conclusion is that the GMO variety is not significantly different from the reference varieties and equivalence is more likely than not (outcome types 1-4). Second, a stronger conclusion can be given and the null hypothesis of non-equivalence will be rejected (in favour of the alternative hypothesis of equivalence) when the confidence interval bar falls entirely within the adjusted equivalence limit lines (outcome types 1 and 2). Finally, when the confidence interval bar lies completely outside the adjusted equivalence limits (outcome type 7), the null hypothesis of equivalence can be rejected and the reasonable conclusion is that of non-equivalence.

The interpretation of the outcome types 3-6 with respect to GMO risk assessment may be more difficult than for types 1, 2 and 7, and may need further safety evaluation, possibly using alternative statistical methods. For example, if differences, even if not statistically significant, were consistent over multiple situations, this could indicate the occurrence of unintended effects. Outcome types 1 or 2 may easily be obtained for characteristics that are stable and precisely measured within each genotype, but that have a large natural variation among reference genotypes. Outcome types 3 or 5 may easily result when the measurement precision or within-genotype stability is low in comparison to the natural variation.

We propose to display as many of the analysed characteristics as is feasible simultaneously, on the same graph (see the Results - Field trial example section). More than one graph is required if characteristics are analysed on different scales, and/or if some are transformed and others not.

### Testing the method by simulation

The performance of difference and equivalence tests was investigated using simulation. The simulation settings were based on the real field trial data (see Methods section for details).

In the first simulation study (reported in Table 1, upper part) the GMO mean was a random draw from the same distribution as the reference varieties, thus mimicking a stochastic equivalence between GMO and reference varieties. When the counterpart mean was chosen equal to the GMO mean, the theoretical size of the difference test (0.1) was reproduced. When the counterpart mean was a random draw from the reference distribution or was set equal to the reference mean, there were almost always true differences between GMO and counterpart, and these were detected with a power around 80%. The performance of the equivalence tests was independent of the choice of the counterpart mean. Equivalence was detected with 95% probability using test E1 and with 76-77% probability using test E2. Non-equivalence was detected with less than 1% probability in test E3.

**Table 1:** Simulation results for various choices of true GMO mean M_G _and true counterpart mean M_C_.

M_G_	M_C_	power test D	power test E1	power test E2	power test E3
~ N(M_R_, V_g_)	~ N(M_R_, V_g_)	0.8289	**0.9485**	0.7603	0.0076
~ N(M_R_, V_g_)	= M_R _(= 0)	0.7587	**0.9481**	0.7668	0.0091
~ N(M_R_, V_g_)	= M_G_	**0.1024**	**0.9496**	0.7704	0.0076

M_R_+LSD(GR;2;95)	~ N(M_R_, V_g_)	0.9724	**0.4957**	**0.0524**	**0.0523**
M_R_+LSD(GR;2;95)	= M_R _(= 0)	1.0000	**0.4964**	**0.0551**	**0.0537**
M_R_+LSD(GR;2;95)	= M_G_	**0.1006**	**0.4954**	**0.0514**	**0.0509**

True reference mean M_R _= 0 in all cases. "~ N(M_R_, V_g_)" refers to a random draw from a normal distribution with the specified mean and variance. LSD(GR;2;95) refers to the theoretical least significant difference calculated using equation 3 with theoretical variances and infinite degrees of freedom. Number of iterations was 10,000. Values in bold can be compared to theoretical values (0.1, 0.95, 0.5 or 0.05).

In the second simulation study (reported in Table 1, lower part) the GMO was chosen at the border between equivalence and non-equivalence, M_R_+LSD(GR;2;95) (see Methods section for details). The border value 0.2407 on ln scale corresponds to a relative difference between GMO mean and reference mean of 100*[exp(0.2407)-1] = 27.2%. As in the first study the theoretical size of the difference test (0.1) was reproduced when the counterpart mean was chosen equal to the GMO mean, and again, the results for the equivalence tests did not depend on the choice of the counterpart mean. Under this null-hypothesis both the proof of equivalence test (E2) and the proof of non-equivalence test (E3) were rejected in about 5% of the simulations, which is the theoretical size of the tests. The shared risk test (E1) accepted equivalence in 50% of the simulations, as expected.

**Figure 2:**
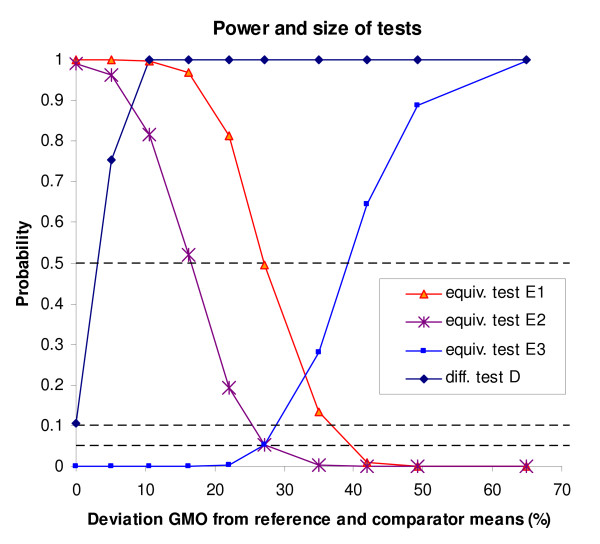
**Results of the simulation study**. Power of difference and equivalence tests as a function of relative difference *dif *= 100*[exp(*d*)-1], where *d *is the difference on ln scale. For *dif *= 0 the reported power is also the size of the difference test D. For *dif *= 27.2% the reported power is also the size of equivalence tests E2 and E3.

The results of the third simulation series are shown in Figure 2. Here the GMO mean was varied systematically, deviating between *dif *= 0% and *dif *= 65% from the reference mean, and the counterpart mean was set equal to the reference mean. For the case *dif *= 0% the size of the difference test (0.10) is reproduced, in all other cases there are true differences, and it can be seen that the power of test D quickly rises to effectively 100% at *dif *= 10%. Figure 2 also shows the behaviour of the three equivalence tests. The proof of equivalence and proof of non-equivalence tests (tests E2 and E3, respectively) are seen to have the nominal size (0.05) at the equivalence/non-equivalence borderline value *dif *= 27.2%. The equivalence test based on testing the difference between GMO and reference varieties, resulting in a statement whether (non-) equivalence is more likely than not (test E1) was not designed as a real proof of equivalence test. As expected, test E1 has a probability of 50% to conclude equivalence for *dif *= 27.2%. In comparison to test E2 it has higher power to find true equivalence, but of course pays for this by also having a larger probability to state 'equivalence more likely than not' when in fact there is non-equivalence. Finally, it can be noted that between 5% and 25% the GMO is still often found to be equivalent to the reference varieties, although it is very likely that at the same time a significant difference with the conventional counterpart is found.

### Field trial example

**Figure 3:**
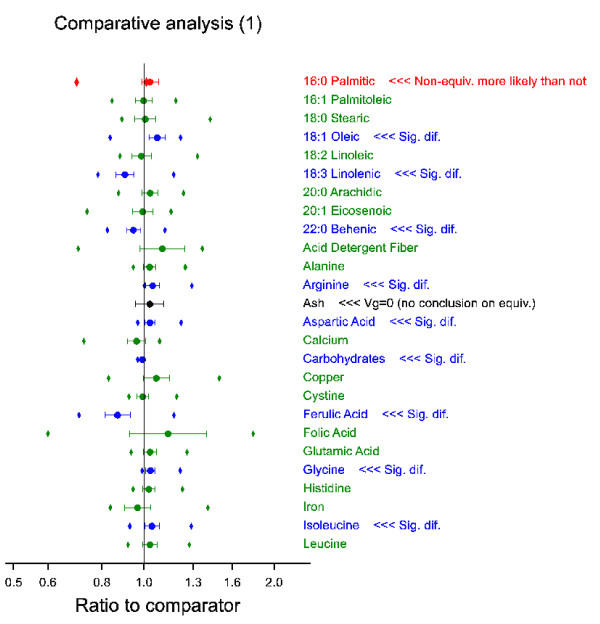
**Part 1 of example comparative analysis**. Circles and bars represent point estimate and 90% confidence interval for ratio GMO to counterpart (here termed comparator). Diamonds represent adjusted equivalence limits based on reference varieties. Colours represent different types of outcome, cf. Figure 1. Green: 1; Blue: 2; Black: 3-4 and cases with genotype variance (Vg) estimated zero; Red: 5-7.

**Figure 4:**
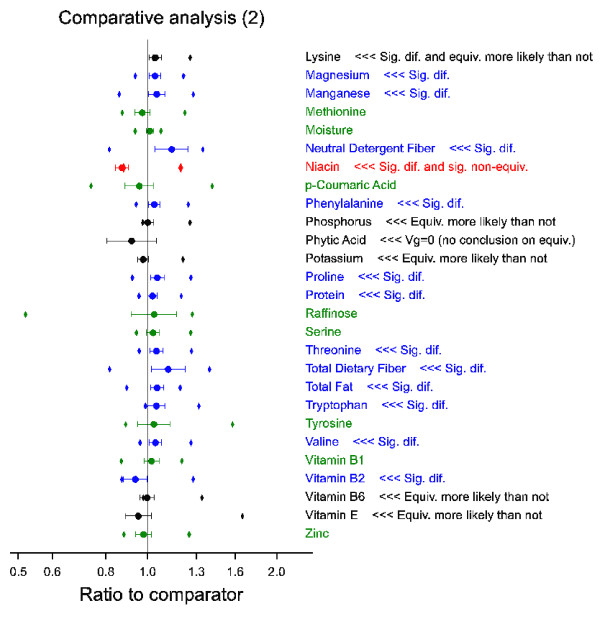
**Part 2 of example comparative analysis**. Circles and bars represent point estimate and 90% confidence interval for ratio GMO to counterpart (here termed comparator). Diamonds represent adjusted equivalence limits based on reference varieties. Colours represent different types of outcome, cf. Figure 1. Green: 1; Blue: 2; Black: 3-4 and cases with genotype variance (Vg) estimated zero; Red: 5-7.

**Figure 5:**
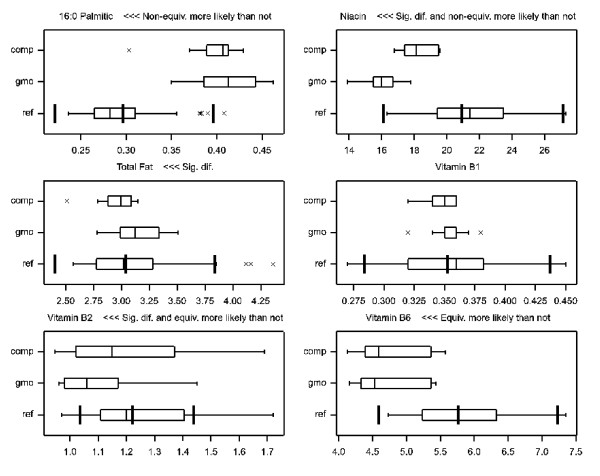
**Boxplot examples for selected analytes **[24]. Each box extends from the lower to the upper quartile (p25 to p75) and the line in the middle is the median (p50). The whiskers extend to extreme data points (minimum and maximum), unless points are farther away from the quartiles than 1.5 times the box length, in which case the points are shown separately as crosses and the whiskers only cover the remaining points. comp = comparator (conventional counterpart); gmo = GMO; ref = reference varieties. Additional thicker bars in the boxplot for references represent geometric mean and calculated equivalence limits.

The proposed methods are illustrated by an example using real data provided by EFSA. Since this paper is not intended to contribute to the risk assessment of specific cases, the data are presented anonymously (see Methods section for details). The precise calculations are described in the Methods section. A graphical overview of the results of the comparative analysis is shown in Figures 3 and 4. More detailed results are given in Figure 5, and in Tables 2 and 3.

**Table 2:** Geometric means for counterpart (Gmc = exp(*m*_*C*_)), GMO (Gmgmo = exp(*m*_*G*_)) and reference varieties (Gmref = exp(*m_R_*)), and variance components for random terms in mixed model: genotype (*Varg*), site (*Vars*), replication within site (*Varr*) and residual (*Var0*).

Analyte	Gmc	Gmgmo	Gmref	Varg	Vars	Varr	Var0
16:0 Palmitic	0.396	0.409	0.296	0.01573	0.003597	0	0.003831
16:1 Palmitoleic	0.004	0.004	0.004	0.00982	0	0.000873	0.003754
18:0 Stearic	0.047	0.047	0.056	0.0274	0.000701	0.000814	0.00554
18:1 Oleic	0.871	0.935	0.812	0.03305	0.000298	0.000307	0.003484
18:2 Linoleic	1.512	1.492	1.675	0.01189	0.002407	0.000909	0.004923
18:3 Linolenic	0.034	0.031	0.034	0.01336	0.003134	0.00088	0.004658
20:0 Arachidic	0.013	0.013	0.013	0.01037	0.001517	0.000802	0.003484
20:1 Eicosenoic	0.011	0.011	0.01	0.01846	0.003502	0.000642	0.005708
22:0 Behenic	0.005	0.005	0.005	0.01075	0.002956	0.00046	0.002557
Acid Detergent Fiber	3.52	3.884	3.523	0.0071	0.000355	0	0.027063
Alanine	6.172	6.366	6.999	0.01215	0.001757	0.001137	0.001974
Arginine	3.641	3.816	4.153	0.00264	0.00029	0.000318	0.002505
Ash	1.13	1.167	1.27	0	0.005899	0.000455	0.010976
Aspartic acid	5.281	5.453	5.967	0.00774	0.000515	0.000592	0.001391
Calcium	51.015	49.108	42.441	0.02556	0.012244	0	0.004253
Carbohydrates	75.683	75.084	74.458	0.00006	0.000177	6E-07	0.000041
Copper	1.161	1.242	1.322	0.03896	0	0	0.009164
Cystine	1.699	1.689	1.819	0.00544	0.000305	0.001014	0.001862
Ferulic acid	2008	1747	1841	0.01331	0.00426	0.000544	0.008106
Folic acid	0.543	0.618	0.573	0.01341	0	0.001377	0.079694
Glutamic acid	15.536	16.056	17.57	0.01474	0.001769	0.00147	0.00226
Glycine	3.063	3.172	3.395	0.00243	0.00023	0.00041	0.001327
Histidine	2.389	2.452	2.63	0.00656	0	0.000705	0.001949
Iron	17.11	16.539	18.846	0.01445	0.01215	0	0.009386
Isoleucine	2.747	2.869	3.088	0.01289	0.000131	0.00151	0.00289
Leucine	10.231	10.562	11.57	0.01851	0.004143	0.001788	0.002688
Lysine	2.602	2.715	2.925	0.00074	0.00018	0	0.002259
Magnesium	1060	1104	1144	0.00672	0.000345	0.000045	0.001882
Manganese	6.377	6.705	6.67	0.03391	0.002448	0.000885	0.003924
Methionine	1.767	1.718	1.889	0.01255	0.001974	0.001786	0.003079
Moisture	11.94	12.093	11.973	0.00098	0.007057	0	0.000693
Neutral detergent fiber	8.629	9.826	9.166	0.00409	0.00175	0.001613	0.015329
Niacin	18.241	15.9	20.915	0.01264	0.002064	0.000351	0.002513
p-coumaric acid	154.5	147.9	165.5	0.05678	0.00603	3.46E-05	0.011303
Phenylalanine	4.102	4.255	4.631	0.0145	0.002208	0.001255	0.001975
Phosphorus	2799	2804	3177	0.00427	0.002569	0	0.002005
Phytic acid	0.57	0.523	0.658	0	0.000268	0.009507	0.031919
Potassium	3242	3171	3604	0.00313	0.008	0	0.001503
Proline	7.29	7.69	7.997	0.01372	0	0.002004	0.002592
Protein	8.222	8.449	9.132	0.00878	0.000437	0.000713	0.001314
Raffinose	0.113	0.118	0.09	0.04279	0.017792	0	0.028393
Serine	4.119	4.242	4.628	0.00901	0.003357	0.00139	0.002331
Threonine	2.724	2.858	3.046	0.00621	0	0.000773	0.002362
Total dietary fiber	11.448	12.801	12.301	0.00545	0.00252	0.000949	0.015822
Total fat	2.979	3.138	3.038	0.01024	0.001744	0.000382	0.002228
Tryptophan	0.481	0.505	0.543	0.00176	0.000333	0.000318	0.004406
Tyrosine	2.674	2.768	3.152	0.01124	0.003216	0.000612	0.01474
Valine	3.739	3.902	4.239	0.00793	0	0.000922	0.002064
Vitamin B1	0.344	0.352	0.352	0.00844	0.00015	0.000611	0.003195
Vitamin B2	1.177	1.103	1.221	0.00269	0.018009	0	0.007777
Vitamin B6	4.736	4.723	5.764	0.00946	0.009281	0.000296	0.002828
Vitamin E	0.006	0.006	0.009	0.02613	0.000318	0.000115	0.009676
Zinc	19.535	19.111	21.338	0.0136	0.001851	0.000192	0.003364

**Table 3:** Assessment of differences and equivalences.

	GMO vs. counterpart					GMO vs. reference mean				
**Analyte**	**ratio**	**dlow**	**dupp**	**sed_GC;1_**	**df_GC;1_**	**ratio**	**elow**	**eupp**	**sed_GR;2_**	**df_GR;2_**

16:0 Palmitic	1.034	0.9887	1.081	0.02658	49.3	1.3811	1.0317	1.849	0.1317	10.5
16:1 Palmitoleic	0.999	0.9558	1.045	0.02638	38.7	0.9982	0.7841	1.271	0.1055	8.4
18:0 Stearic	1.008	0.9538	1.066	0.03307	41.7	0.8355	0.57	1.225	0.1736	10.9
18:1 Oleic	1.074	1.0294	1.121	0.02538	42.1	1.1519	0.7595	1.747	0.1897	11.2
18:2 Linoleic	0.987	0.9366	1.04	0.03121	42.2	0.8908	0.6886	1.152	0.1155	10
18:3 Linolenic	0.904	0.8606	0.95	0.02942	39.9	0.9234	0.7009	1.216	0.1221	9.1
20:0 Arachidic	1.034	0.9906	1.079	0.02545	41.5	0.9931	0.7816	1.262	0.1075	10
20:1 Eicosenoic	0.995	0.9419	1.051	0.03253	41.4	1.11	0.8068	1.527	0.1432	10
22:0 Behenic	0.946	0.9117	0.981	0.0218	41.4	0.9766	0.7672	1.243	0.1089	10.4
Acid detergent fiber	1.103	0.9805	1.241	0.07046	51.9	1.1026	0.8373	1.452	0.1027	4.4
Alanine	1.031	0.9986	1.065	0.0192	41.4	0.9096	0.7052	1.173	0.1154	10.8
Arginine	1.048	1.0107	1.087	0.02153	41.9	0.9188	0.8077	1.045	0.0558	7.9
Ash	1.033	0.958	1.114	0.04498	54.6	0.9187	0.858^1^	0.984^1^	0.0341	53.6
Aspartic acid	1.033	1.0049	1.061	0.0161	41.4	0.9138	0.7455	1.12	0.0921	10.7
Calcium	0.963	0.9184	1.009	0.02801	48.5	1.1571	0.7993	1.675	0.1673	10.6
Carbohydrates	0.992	0.9875	0.997	0.00274	42	1.0084	0.9895	1.028	0.0083	8.2
Copper	1.069	0.9963	1.148	0.04221	51	0.9395	0.5947	1.484	0.2073	10.8
Cystine	0.994	0.9636	1.026	0.01863	40.1	0.9286	0.7797	1.106	0.0779	9.5
Ferulic acid	0.87	0.8132	0.931	0.04008	41.8	0.9489	0.7179	1.254	0.1234	9
Folic acid	1.138	0.929	1.395	0.12096	44.1	1.0786	0.6948	1.674	0.1515	3.6
Glutamic acid	1.033	0.9984	1.07	0.02055	41.4	0.9138	0.6908	1.209	0.127	10.9
Glycine	1.035	1.0084	1.063	0.01571	40.2	0.9343	0.8292	1.053	0.0525	8.8
Histidine	1.026	0.994	1.06	0.01903	40.7	0.9325	0.7711	1.128	0.0853	10
Iron	0.967	0.9015	1.036	0.04159	49.4	0.8776	0.6544	1.177	0.1288	8.6
Isoleucine	1.044	1.0045	1.086	0.0232	41.6	0.9292	0.7137	1.21	0.1192	10.5
Leucine	1.032	0.9942	1.072	0.02241	41.3	0.9129	0.6673	1.249	0.1422	10.9
Lysine	1.043	1.0084	1.08	0.02037	48.8	0.9282	0.8532	1.01	0.0322	4.7
Magnesium	1.041	1.0089	1.074	0.01863	42.6	0.9647	0.796	1.169	0.0863	10
Manganese	1.051	1.0047	1.1	0.02702	41.1	1.0053	0.6585	1.535	0.1922	11
Methionine	0.972	0.9338	1.012	0.02398	41.1	0.9095	0.7004	1.181	0.1177	10.3
Moisture	1.013	0.9938	1.032	0.0113	49.5	1.01	0.9357	1.09	0.0336	8.6
Neutral detergent fiber	1.139	1.0413	1.245	0.05322	43.2	1.072	0.8703	1.32	0.0778	4.4
Niacin	0.872	0.8405	0.904	0.0216	41.7	0.7602	0.5862	0.986	0.1178	10.8
p-coumaric acid	0.957	0.8867	1.034	0.04565	42.1	0.8938	0.5145	1.553	0.2498	10.6
Phenylalanine	1.037	1.0043	1.071	0.01921	41.4	0.9187	0.6965	1.212	0.1258	11
Phosphorus	1.002	0.9698	1.034	0.01923	48.5	0.8823	0.7537	1.033	0.0694	8.8
Phytic acid	0.919	0.8042	1.049	0.07949	53.2	0.7958	0.7082^1^	0.894^1^	0.0581	52.2
Potassium	0.978	0.9509	1.006	0.01665	47.9	0.8798	0.7675	1.009	0.0595	8.2
Proline	1.055	1.0166	1.095	0.02199	41.9	0.9616	0.7333	1.261	0.1227	10.7
Protein	1.028	1.001	1.055	0.01566	41.2	0.9252	0.7454	1.148	0.098	10.8
Raffinose	1.036	0.9177	1.17	0.07232	49.1	1.3123	0.7939	2.169	0.2218	8.9
Serine	1.03	0.9944	1.067	0.02087	41.1	0.9167	0.7343	1.144	0.0998	10.2
Threonine	1.049	1.0131	1.087	0.02094	41.5	0.9383	0.7788	1.13	0.0834	9.8
Total dietary fiber	1.118	1.0211	1.225	0.05404	41.2	1.0407	0.8284	1.307	0.087	4.7
Total fat	1.054	1.0181	1.09	0.02035	41.9	1.033	0.8173	1.306	0.1062	10.8
Tryptophan	1.049	1.0002	1.101	0.02851	44	0.9292	0.8205	1.052	0.0487	5.1
Tyrosine	1.035	0.9483	1.13	0.05215	44.4	0.8782	0.6674	1.156	0.117	7.3
Valine	1.043	1.0096	1.078	0.0196	41.8	0.9203	0.7477	1.133	0.0936	10.3
Vitamin B1	1.023	0.9818	1.065	0.02433	43.3	0.9999	0.8059	1.241	0.0972	10.3
Vitamin B2	0.938	0.8799	0.999	0.03784	49	0.9036	0.7671	1.064	0.0611	4.4
Vitamin B6	0.997	0.9597	1.037	0.0229	41.6	0.8194	0.6529	1.028	0.1025	10.4
Vitamin E	0.953	0.8874	1.023	0.04217	42.5	0.6908	0.4718	1.011	0.1709	9.9
Zinc	0.978	0.9381	1.02	0.02495	42.5	0.8956	0.6814	1.177	0.1225	9.9

The 90% confidence limits (dlow, dupp) are expressed on the scale of ratio GMO to counterpart mean. *sed*s are on ln scale, ratio and 90% confidence limits are back-transformed. The 95% equivalence limits (elow and eupp) calculated on the scale of the ratio of GMO to reference mean. The point estimate of this ratio itself is given in the column ratio. The width of the equivalence interval depends on *sed*_GR;2 _, given on the logarithmic scale, and the degrees of freedom for equivalence (df_GR;2_) calculated by the Kenward-Roger method. See text for further explanation.

^1 ^For ash and phytic acid the equivalence intervals are not trustworthy, because the estimate of the variance between reference genotypes was 0 and *sed*_*GR;2 *_is based on lower strata (note also the high values of *df*_*GR;2 *_).

Figures 3 and 4 show the relative differences of the GMO with respect to the conventional counterpart for 53 plant characteristics. For example, relative large deviations are seen for acid detergent fiber (+10%), ferulic acid (-13%), folic acid (+14%), neutral detergent fiber (+14%), niacin (-13%) and total dietary fiber (+12%). However, depending on the variability and uncertainty underlying them, large differences may not be statistically significant (e.g. the interval for acid detergent fiber includes 1, so the difference is not significant), while smaller differences may be (e.g. glycin is significantly higher in the GMO than in the counterpart, with a point estimate of only +3.5%). Note that the significance tests are based on a standard error of difference (see Table 3) which is calculated from the residual variance *V*_ε _(see Table 2) as $sed_{GC;1}=\sqrt{V_{\varepsilon }\left(\frac{1}{12}+\frac{1}{10}\right)}$, where 12 and 10 are the number of replications in this experiment for GMO and counterpart, respectively. The number of degrees of freedom, estimated by the Kenward-Roger method, varies between 38.7 (16:1 palmitoleic) and 54.6 (ash).

In total there were twenty-three analytes with a significant difference between GMO and counterpart (which is 43% of the 53 investigated analytes). These analytes are shown in blue (or in black or red if there was also a potential equivalence problem) in Figures 3 and 4, and some examples of boxplot representations [24] of these data are shown in Figure 5 to assist further interpretation. Note, however, that these boxplot representations ignore some aspects of the model, such as site and replication variation. Therefore, the boxplots alone cannot provide a complete overview.

The variation between reference varieties has been used to calculate equivalence limits. Although conceptually there is just one set of equivalence limits, the limits were calculated on three different scales. Each scale is useful for a specific purpose.

1. The first scale is the natural scale which allows food/feed experts to recognize values most easily. For instance, niacin has an equivalence interval [16.1, 27.1] when back transformed on this natural scale. These intervals are shown in the boxplots (Figure 5).

2. The second scale is the ratio scale where the GMO is compared to the mean of the reference varieties (see Table 3). This scale provides the most direct view whether the difference between GMO and references is significant (it is significant if the interval does not contain the value 1). This scale is therefore best for distinguishing between equivalence categories (ii) and (iii) (test E1). For niacin the equivalence interval on this scale is [0.59, 0.99], so indeed the difference is significant and non-equivalence is more likely than not.

3. Finally, the equivalence interval can be expressed on the adjusted scale where both GMO and references are compared to the conventional counterpart (see Figures 3 and 4); this scale allows a simultaneous presentation of the results for both the comparison of GMO with the counterpart and the comparison of GMO with the reference lines. Therefore it is the easiest scale for performing a test of equivalence by the graphical equivalent of the TOST procedure advocated in this paper (see Figures 3 and 4). For the example of niacin the equivalence interval on this adjusted scale is [0.88, 1.20]. This interval overlaps with the confidence interval for the comparison of the GMO with its counterpart (which is [0.84, 0.90], see Table 3), therefore neither equivalence nor non-equivalence has been proven for this analyte (tests E2 and E3).

In any case, the three intervals are just adjusted versions of each other and completely equivalent for statistical testing as explained more fully in the Method section. In the current example two cases were found where, on applying test E1 the GMO point estimate falls outside the calculated equivalence limits, or, in other words, there was a statistically significant difference between the GMO and the references (16:0 palmitic and niacin). For these analytes non-equivalence is more likely than not. For further interpretation boxplots are given in Figure 5. It can be seen that for 16:0 palmitic both the GMO and the counterpart are higher than the reference range, therefore on this single characteristic GMO and counterpart seem to present the same hazards, if any. It is outside the scope of this document to discuss the risk assessment of such cases. For niacin the situation is different. Niacin is found 24% lower in the GMO than on average in the reference varieties, and the result is also significantly lower (by 13%) than what is found for the counterpart.

A problem occurs when the variance component between reference genotypes is estimated as zero. In the current example dataset this occurred with ash and phytic acid. In these cases the calculation of standard errors of difference will be based on the assumption that there is no variation between the reference genotypes, and standard errors and degrees of freedom are derived from a model which omits the random factor for genotypes. This is not a truly believable model: too many degrees of freedom will be assigned to the standard error of difference (see Table 3) leading to equivalence intervals which are typically too narrow.

Accepting the calculated equivalence limits as null hypothesis values in a test of equivalence for the remaining 49 analytes leads to the conclusion that 44 are proven to be equivalent to the reference varieties, whereas for 5 (lysine, phosphorus, potassium, vitamin B6 and vitamin E) the equivalence is more likely than not, but not strictly proven at the 95% confidence level. For further interpretation boxplots can be given, see examples in Figure 5.

## Discussion

Difference testing and equivalence testing can both contribute to a meaningful comparative assessment. First, the GMO can be different from its appropriate non-GM counterpart, and a difference may constitute a hazard (or potential risk) which should be subject to further safety evaluation. This is why the proof of difference is sometimes referred to as "proof of hazard", but this is a misleading term because there are many differences that present no hazard for human health. Secondly, a GMO can be equivalent to appropriate references, such as a range of commercial varieties. Established equivalence of a GMO has been interpreted as relevant for subsequent toxicological risk assessments. It should be stressed that statistical approaches should never be used for automatic decisions of food safety, but as tools providing the appropriate context for the final safety assessement.

For testing of differences and equivalences two-sided tests (both increased and decreased characteristics are relevant) is the most common case, but if it is *a priori *known that differences can only be in one direction, then it can be easily adapted to one-sided versions (looking only at increases or decreases).

Not always will there be datasets from field trials with reference varieties in their trial design. Use of literature data may occasionally be considered as an alternative source for quantifying background variation, but may present great difficulties both regarding the representativity of the data and the possibilities to discern the different components of variation. Further discussion is given in [11].

We described how the simultaneous application of difference and equivalence testing can lead to seven possible types of outcome (see Figure 1). With respect to the necessity of further evaluation to assess a possible impact of GMOs on human/animal health, the patterns of significant differences (Types 2, 4, 6, 7) should be inspected for biologically relevant signals. Cases with a clearly established non-equivalence in test E3 (Type 7) and cases where non-equivalence is found more likely than not in test E1 (Types 5, 6) require further evaluation. Risk assessors may also require further evaluation for cases where equivalence is more likely than not according to test E1, but not significant in the formal equivalence test E2 (Types 3, 4). Risk characterization will then be used by assessors to specify what further evaluation is needed, based on considerations linked to patterns of observed results and biological or toxicological relevance.

Experiments should be designed to have sufficient statistical power to be able to reject the null hypotheses being tested for relevant magnitudes of effect. However, the use of observed power, which is power estimated from the data arising from the experiment itself, is not a valid alternative. It has been proposed that equivalence can be concluded for a non-significant difference, provided that the observed power of the difference test for a difference at the equivalence limit is at a specified high level. However, Tempelman [25] pointed out how with those criteria a poorly executed experiment would be rewarded a greater chance of concluding equivalence than an experiment with a better precision. Power analysis must therefore be done prior to the experiment.

It can be noted that for difference and equivalence testing approaches power analysis has a different purpose. First, risk assessors should require that a difference test will find true differences of a specified magnitude in a substantial probability of cases (e.g. 80%). Secondly, applicants for introducing a GMO on the market have an interest that a truly equivalent GMO will pass the equivalence test with high probability. This requires a power analysis where the relevant effect level is for example a zero or small difference between the GMO and the mean of the reference varieties.

In this paper the focus is on comparing characteristics averaged over environments. In the biomedical literature on equivalence testing this is known as an approach for average (bio)equivalence. Alternative approaches are based on the idea of individual (bio)equivalence related to the question of switchability of the treatments [12]. In the context of field trials this can be translated to requiring equivalence in the population of environments (sites). In the linear mixed model approach the genotype by environment interaction would have to be estimated [26], which is typically easy to do (see Methods section for details). However, the consequences for safety assessment are still unclear, for example would it be possible to declare a GMO equivalent in some environments and not in others? More discussion on such issues is needed before a statistical approach can be devised.

Commercial reference varieties have also been included in animal feeding studies (e.g. [8,12,27,28]). In principle our proposed method can be used there as well. However, when it is expected that the investigated characteristics (such as animal blood and urine parameters) do not vary at all between reference varieties used in the feed, this would not conform to the basic idea proposed here of using observed variation between genotypes as a basis to determine equivalence limits. Further research on such cases is needed.

The method proposed in this paper may contribute to an objective and transparent process of risk assessment. However, several issues remain to be solved. First, the approach should be adapted for data which cannot readily be transformed to normality, such as counts, quantal or ordinal data. Second, research is needed for the power analysis of mixed model tests. More research is needed to characterize the coverage probability of the estimated confidence intervals for small sample sizes, such as three plots, two years, and four sites, because the available models are asymptotic. Moreover, research is needed for an optimal design, i.e. optimal numbers of plots and sites for a most powerful decision on equivalence. Statistical analysis may need to be adapted to more complicated designs (e.g. repeated measures). And last but not least, these methods may be adapted to the simultaneous assessment for multiple characteristics. When performing many simultaneous tests spurious significant results can be expected both in proof of difference and proof of equivalence. There is little experience with multivariate tests of equivalence (see e.g. [29-31]). Multivariate analysis may give an alternative approach to the multiplicity issue. Although some discussion of these issues is given in [11], more research is needed.

## Conclusions

Safety assessment of GMOs is ultimately a complex undertaking in which the interpretation of compositional data is only one element. And even in this restricted setting the role of statistical methodology is limited to provide a context for the final biological interpretation of results. Nevertheless, this interpretation can benefit from a standardised statistical approach that clearly shows differences and equivalences in a comparable manner.

The main purposes of the comparative assessment are twofold: to demonstrate whether the GMO and/or derived food/feed is different from its appropriate non-GM counterpart and/or to demonstrate whether it is equivalent to appropriate reference varieties, apart from the intended changes. This paper proposes a statistical methodology that is not focussed exclusively on either differences or equivalences, but that provides a richer framework within which the conclusions of both types of assessment are allowed simultaneously. The approaches are complementary: statistically significant differences may point at biological changes caused by the genetic modification, but which are not relevant from the viewpoint of food safety. On the other hand, equivalence assessments are used to classify differences as being inside or outside the range of natural variation. A procedure combining both approaches will aid the subsequent interpretation of the statistical results.

A simulation study using realistic variance values validated the expected probabilities of the tests proposed. An important conclusion is in a typical situation of variabilities a range of deviations exists, say between 5% and 25%, where the GMO is still equivalent to the reference varieties, although it is very likely to find a significant difference with the conventional counterpart. This illustrates that the application of equivalence testing is a useful complement to the traditional practice of performing difference tests.

The conclusions drawn for the example field trial dataset can be summarised as follows. For 23 out of 53 analytes there were statistically significant differences (at the 90% confidence level) between GMO and counterpart. The differences varied between -13% and +14%. For two analytes, 16:0 palmitic and niacin, a statistically significant deviation (at the 95% confidence level) from the mean of the reference varieties was found, and non-equivalence was more likely than not. Further evaluation is required. For five analytes, lysine, phosphorus, potassium, vitamin B6 and vitamin E, equivalence was more likely than not, but a strict proof of equivalence cannot be given. Further evaluation may be required. For two analytes, ash and phytic acid, no proper conclusion on equivalence can be formulated because of lack of observable natural variation in the reference varieties. Further evaluation may be required. For 44 analytes (including 20 with significant differences between GMO and counterpart) equivalence was established in a formal test of equivalence (at the 95% confidence level) using the estimated equivalence limits.

## Methods

### Linear mixed models

Measurements can be made on several scales (continuous, ordinal, quantal, binary, count, multinomial). Here, we focus on the continuous scale, which is appropriate for most compositional, agronomic and phenotypic variables in field studies. For measurements made on other scales it is often possible to devise similar statistical approaches to those described here.

It is often appropriate to transform data before standard statistical methods are used. For example, many biological effects are better described as multiplicative rather than additive effects. Differences are commonly expressed as a percent change, i.e. as relative differences (ratios) rather than absolute differences. On the other hand, most statistical models are additive models for estimating or testing absolute differences. A logarithmic transformation of the data may be appropriate because it transforms a multiplicative model into an additive model, and thus relative differences into absolute differences through the equation log(*A*/*B*) = log(*A*)- log(*B*). Only when reporting results (graphs, tables) can these be back-transformed to the original scale. Here, we use logarithmic transformations, but the appropriateness of this should be investigated on a case-by-case basis for other data.

Field experiments are usually replicated at multiple sites. At each site a field trial is conducted with the varieties randomised over plots in multiple blocks (or replications). The statistical analysis of data from the experiments for comparative risk assessment is here restricted to studying the average difference and the average equivalence over sites. The primary objective for an average difference/equivalence approach is neither the identification of possible interactions nor per-site (per-year) evaluation. Instead, overall (for all sites, plots, years) confidence limits are estimated, allowing statements on overall differences and equivalences.

A linear mixed model is used for the statistical analysis of the data set (all sites and/or years) where the factors site and, if present, year are assumed to be either random or fixed, depending on the details of the experimental design. In this paper we assume random site effects. The between-site, between-replicate, between-plot and possibly the between-year variability will be estimated as related variance components.

For the purpose of modelling the differences and similarities from an experimental design with GMO, counterpart and a range of reference varieties, it is useful to describe the genotypes of the measured samples not just by one factor, but by two factors and a dummy variable:

1. *GenotypeGroup*: a 3-level fixed factor distinguishing GMO, conventional counterpart and the group of reference varieties as a whole; note that this factor includes the specific contrasts of interest between GMO and its counterpart, and between GMO and the group of references;

2. *Genotype*: a random factor with as many levels as there are varieties (GMO, conventional counterpart and each of the reference varieties);

3. *IndRef*: an indicator variable with value 1 for the reference varieties, and 0 otherwise.

By including the interaction of *Genotype *and the (uncentered) indicator variable *IndRef *in a mixed model, the GMO and its counterpart are not considered as levels of the random factor. Therefore in such a model the difference between the GMO and its counterpart and the difference between the GMO and the mean of the reference varieties, which are both specific contrasts of the fixed factor *GenotypeGroup*, will be assessed against the proper residual variation, excluding the variance between genotypes. If on the other hand the indicator variable is omitted the residual variation will include the variance between reference genotypes which is appropriate for establishing equivalence limits from the full range of reference varieties. Note that in neither of these models do the GMO and counterpart means contribute to the residual variance component because of the presence of the fixed factor *GenotypeGroup*. In the case of the example field trial data the ln transformed data were thus analysed with two slightly different versions of the mixed model, hereafter named model 1 and model 2, respectively. For the tests of difference and equivalence:

(1) yijkl=Mean+Sitei+Blockij+GenotypeGroupk+IndRefl⋅Genotypel+εijkl

and for establishing equivalence limits:

(2) $$\begin{array}{c} y_{ijkl}=Mean+Site_{i}+Block_{ij}+\\ GenotypeGroup_{k}+Genotype_{l}+\varepsilon_{ijkl} \end{array}$$

where *i*, *j*, *k *and *l *are indices for site, block within site, treatment group (counterpart, GMO or reference) and reference varieties, respectively. The response *y_ijkl _*is the log-transformed result, using the natural logarithm (ln). The fixed factors in this model are *Mean*, the overall mean, and *GenotypeGroup_k_*, the average deviation from the overall mean for each of the three treatment groups (*k *= 1: counterpart, 2: GMO, 3: reference genotypes). The random factors in the model are *Site_i_*, the average deviation for site *i*, *Block_ij_*, the average deviation for block *j *of site *i*, *Genotype_l_*, the average deviation for reference genotype *l*, and *ε_ijkl_*, the residual term for each measurement. As usual in mixed modelling, the random terms are assumed to arise independently from normal distributions with mean 0 and a certain variance component that is to be estimated (*V_s_*, *V_b_, V_g _*and *V_ε_*, respectively). A common way to fit mixed models to data is the residual maximum likelihood (REML) algorithm, which is available in all major statistical packages.

If it would be needed in addition to the average difference/equivalence approach of this paper, the site by genotype interaction can be investigated by defining another indicator variable, with value 0 for the reference varieties, and 1 otherwise, and including its interaction with *GenotypeGroup *as an additional fixed effect (uncentered) in the model. We do not further pursue this model here (see Discussion section).

For comparing all tested varieties we need estimated means, *m*_*C *_,*m*_*G *_and *m*_*R *_(for counterpart, GMO and references, respectively) and the standard errors of difference, *sed*_*GC;i *_and *sed*_*GR;i*_, where *i *is the model adopted.

Where as these estimates are easily available in mixed models from standard software, they can also be written out explicitly for both model 1 and model 2, at least when the datasets are balanced (GMO, counterpart and *n_g _*reference genotypes in *n*_*r *_replicates on *n*_*s *_sites):

(3a) $$sed_{GC;1}=\sqrt{2\frac{V_{\varepsilon }}{n_{s}n_{r}}}$$

(3b) $$sed_{GC;2}=\sqrt{2\left(V_{g}+\frac{V_{\varepsilon }}{n_{s}n_{r}}\right)}$$

(3c) $$sed_{GR;1}=\sqrt{\frac{V_{\varepsilon }}{n_{s}n_{r}}+\frac{V_{g}}{n_{g}}+\frac{V_{\varepsilon }}{n_{g}n_{s}n_{r}}}$$

(3d) $$sed_{GR;2}=\sqrt{V_{g}+\frac{V_{\varepsilon }}{n_{s}n_{r}}+\frac{V_{g}}{n_{g}}+\frac{V_{\varepsilon }}{n_{g}n_{s}n_{r}}}$$

Approximate two-sided 100(1-α)% confidence intervals are based on the *sed*s and *t_df_*_;_*_p _*, being the *p *= 100(1-α/2)% point of the appropriate Student's *t *distribution, where *df *is the appropriate number of degrees of freedom. For the calculation of *df *the method of Kenward and Roger [32] has been recommended [33]. The product *t_df_*_;1-_*_α_*_/2 _*sed *is called the least significant difference (*lsd*) and may be obtained directly in some statistical packages. For the comparison between test materials X and Y using model *m *(1 or 2) and one-sided confidence percentage *p *we will denote this by *lsd(XY;m;p)*.

The appropriate calculations to perform the difference and equivalence tests are as follows:

1. For the test of difference (test D) calculate 90% confidence limits as *m_G _± lsd*(*GC*;1;95); when the counterpart mean *m_C _*falls outside this confidence interval the difference between GMO and counterpart is statistically significant.

2. For the equivalence tests, calculate equivalence limits *EL_L _*and *EL_U _*as 95% confidence limits around the reference mean *m_R _*: *m_R _± lsd*(*GC*;2;97.5).

3. When the GMO mean *m_G _*falls outside the equivalence interval [*EL_L_*, *EL_U_*], the difference between the GMO and the reference variety group is statistically significant allowing for the background variation between genotypes, and non-equivalence is more likely than not ('shared risk' test of equivalence E1).

4. For the tests of equivalence E2 ('proof of equivalence') and E3 ('proof of non-equivalence') calculate 90% confidence limits as *m_G _± lsd*(*GC*;1;95). Equivalence is proved using test E2 if this confidence interval falls entirely within the equivalence interval [*EL_L_*, *EL*_*U*_]. Non-equivalence is proved using test E3 if the confidence interval falls entirely outside the equivalence interval [*EL*_*L*_, *EL*_*U*_].

All tests are performed on the logarithmic scale. For interpretation of the numerical values, the means and differences of means on the logarithmic scale can be back-transformed to geometric means and ratios of geometric means on the original scale. So, based on a difference *D*, the point estimate of the corresponding ratio is *e^D^*, and the approximate 100(1-α)% confidence interval is *e^D-lsd^*, *e^D+lsd^*.

The practical implementation in two major software packages for assessing the difference of the GMO to the counterpart using model 1 (test D) is as follows (assuming levels 1, 2 and 3 of the *genotypegroup *factor corresponding to counterpart, GMO and references, respectively):

Genstat:

FACTOR [labels=!T(compGMO,ref)] ref_aside

CALC ref_aside = 1*(genotypegroup.in.!(1,2))+\

                 2*(genotypegroup == 3)

VCOMPONENTS\

   [fixed = ref_aside/genotypegroup; cadjust = none]\

   random = site + site.rep + genotype.indref;\

   constraint = pos

REML y

SAS:

proc mixed data = example CL = WALD alpha = 0.1;

class site rep genotype genotypegroup;

model y = genotypegroup/s

      covb outp = out ddfm = kenwardroger;

random site site*rep indref*genotype;

estimate 'gmo_comp' genotypegroup -1 1 0/CL;

run;

The practical implementation for estimating the equivalence limits and for performing equivalence test E1 using model 2 is as follows:

Genstat:

FACTOR [labels=!T(comp,GMOref)] comp_aside

CALC comp_aside = 1*(genotypegroup == 1)+\

                  2*(genotypegroup.in.!(2,3))

VCOMPONENTS\

   [fixed = comp_aside/genotypegroup]\

   random = site + site.rep + genotype;\

   constraint = pos

REML y

SAS:

proc mixed data = example CL = WALD alpha = 0.05;

class site rep genotype genotypegroup;

model y = genotypegroup/s

      covb outp = out ddfm = kenwardroger;

random site site*rep genotype;

estimate 'gmo_ref' genotypegroup 0 1 -1/CL;

run;

For equivalence testing according to tests E2 and E3, again using model 1, the code is as follows:

Genstat:

FACTOR [labels=!T(comp,GMOref)] comp_aside

CALC comp_aside = 1*(genotypegroup == 1)+\

                  2*(genotypegroup.in.!(2,3))

VCOMPONENTS\

   [fixed = comp_aside/genotypegroup; cadjust = none]\

   random = site + site.rep + genotype.indref;\

   constraint = pos

REML y

SAS:

proc mixed data = example CL = WALD alpha = 0.1;

class site rep genotype genotypegroup;

model y = genotypegroup/s

      covb outp = out ddfm = kenwardroger;

random site site*rep indref*genotype;

estimate 'gmo_ref' genotypegroup 0 1 -1/CL;

run;

These program fragments give only the essential central mixed model calculation. Obviously more programming is needed for reading the data, outlier control, data transformation, and post-processing the results to calculate confidence intervals, equivalence limits and plotting the graphs.

The basic information needed from the mixed model are the means, the standard errors of difference and the corresponding degrees of freedom. With the above two specifications of the mixed model (either with *Genotype *or with Genotype. In*dRef *among the random terms) the means and variance components are exactly the same. Only the *sed*s and the *df*s are different. Actually, from equations (3) it follows that the *sed*s from the two models 1 and 2 are related by:

$$\begin{array}{l} {\left(sed_{GC;2}\right)}^{2}={\left(sed_{GC;1}\right)}^{2}+2\cdot V_{g}\\ {\left(sed_{GR;2}\right)}^{2}={\left(sed_{GR;1}\right)}^{2}+V_{g} \end{array}$$

where *V*_*g *_is the variance component estimate for the background variation between the reference varieties. Note that we need the *sed_GC;_*_1 _(so excluding genotypic variation) for the GMO to counterpart difference test D, and the *sed*_*GR*;2 _(including genotypic variation) for estimating the equivalence limits and test E1. For equivalence tests E2 and E3 we need both. Because of the relations fitting either one of the models would be enough to allow the calculation of both *sed*s. The only remaining reason for fitting two models rather than one to the same dataset is the different calculation of the degrees of freedom by the Kenward-Roger method in the two cases.

### Presentation of results

After the appropriate transformation, simultaneous display is facilitated by shifting all relevant values for each particular characteristic to a scale that has *m*_C_, the mean of the conventional counterpart for that characteristic, as its baseline zero value. Therefore, on this new scale, the values of the means of the GMO, its conventional counterpart and the set of reference varieties, become, respectively: *m*_G _- *m*_C_, 0, *m*_R _- *m*_C_. Note that a difference of 0 on an additive scale corresponds to a ratio of 1 on a multiplicative scale. Hence, in principle, for a multiplicative scale, both the mean of the GMO and the equivalence limits can be displayed as ratios to the counterpart (but see below for certain adjustments required to achieve a valid practical outcome).

After shifting all relevant values to the new zero baseline, the confidence limits for the difference test on this new scale become: *m*_G _- *m*_C _± *lsd*(GC;1;95), the equivalence limits *m*_R _- *m*_C _± *lsd*(GR;2;97.5), and the confidence limits for the equivalence tests E2 and E3 *m*_G _- *m*_C _± *lsd*(GR;1;95). Note that the equivalence limits, chosen to be symmetrical around the centre of the distribution of reference varieties, are typically asymmetrical (before and after adjustment) on this new scale. To facilitate visual interpretation, instead of using the two sets of confidence limits in the graphs, it is recommended for convenience that only one be displayed, that for the difference test. Without some adjustment, the confidence limits for the difference test would not give a valid visual representation for the equivalence test on the graph. This problem is overcome by making an adjustment to the displayed equivalence limits. After this adjustment the displayed confidence limits for the difference test may be used as a basis also for the visual representation of the equivalence test. In this way, one confidence limit may serve visually for assessing the outcome of both tests simultaneously. The adjustment of the equivalence limits consists of two steps: (1) scaling the basic equivalence limits, so that the confidence limits required for the difference and equivalence tests have the same width; and (2) an appropriate shift to facilitate display of the adjusted limits, together with m_G_, on the scale that has *m_C _*as its baseline zero value. The adjusted equivalence limits for visual display are calculated by the formula:

$$\left(m_{G}-m_{C}\right)+\left[\left(m_{R}-m_{G}\right)\pm lsd(GR;2;97.5)\right]\cdot \frac{lsd(GC;1;95)}{lsd(GR;1;95)}$$

Note that this adjustment is being made just for the ease of visual display, and that the adjusted confidence limits have exactly the same inclusion probability of 95% for the true difference as the unadjusted confidence interval.

It is recommended that the graph should show the line of zero difference between the GMO and its conventional counterpart and, for each characteristic: the lower and upper adjusted equivalence limits, the mean difference between the GMO and its conventional counterpart, and the confidence limits for this difference (see the set of possible example outcomes for a single characteristic in Figure 1). The horizontal axis is labelled with values that specify the change on the natural scale. In the case of a multiplicative scale and a logarithmic transformation, changes of 2 × and 1/2 × will appear equally spaced on either side of the line of zero difference

### Simulations

Simulation studies were performed to investigate how well the difference and equivalence tests perform. Observations were generated for 8 sites, 4 blocks per site and 8 genotypes (GMO, counterpart and 6 reference varieties, representing commercial varieties in the real world) according to a linear model for logarithmically-transformed observations. The model contained random terms for site, block, genotype and plot (residual) drawn from normal distributions with mean 0 and variances equal to 0.0029, 0.0008, 0.0127 and 0.0073, respectively, which were the average variance components found for 53 analytes in the field trial example (see hereafter). For the 6 reference genotypes independent random deviates were drawn from the genotype distribution with mean 0 and variance 0.0127.

In a first series of simulations the GMO mean was considered exchangeable to the reference genotypes, and therefore its value was set by making a draw from the same normal distribution, with mean 0 and variance 0.0127. Three options for setting the counterpart mean were investigated: a) equivalent to the reference mean (its value was set by making another draw from this same normal distribution); b) equal to the reference mean (its value was set to 0); or c) its value was set identically equal to the GMO mean.

In a second series of simulation the GMO mean was set equal to the theoretical upper equivalence limit calculated as described below. Again the same three options for the counterpart mean were investigated.

In a third series of simulations the counterpart mean was set to be equal to the reference mean (identically equal to 0), and the GMO mean was varied systematically to investigate the power of the tests. Its value was set equal to a value *d *(where *d *was varied systematically between 0 and 0.5 (or, equivalently, the relative difference on the original scale *dif *= 100*[exp(*d*)-1] was varied between 0% and 65%). Note that the choice *d *= 0 corresponds to exact equality of GMO and counterpart, whereas other choices lead to a true difference, though not necessarily non-equivalence, because this depends on what values the equivalence limits are set to.

### Field trial data

The dataset analysed in this paper is an example of a comparative assessment regarding GMO safety using real data from a field study. We consider here 68 compositional characteristics of maize grain. Each characteristic was measured on (i) a GM variety, (ii) a conventional counterpart variety, and (iii) 13 reference varieties. The data come from a randomised block design conducted at four sites in one year. Under the protocol of the experimental design each site was to have been planted with the GM variety, the counterpart variety and four reference varieties in three blocks of six plots, but there were some deviations. The GM variety was replicated three times at each site, and the conventional counterpart variety three times at two of the sites, but only twice at the other two sites. Each of three reference varieties was planted at two sites, but each of the remaining 10 reference varieties at one site only. Most varieties had 3 replicates per site (but in some cases only 2 or even 1). The data analysed here have 14-18 plots per site, for a total of 67 plots. It may be noted that this experimental design is not ideal. For example, the number of sites and the replication per site were relatively low, and the conventional counterpart was not included in all blocks. However, in spite of the shortcomings of the experimental design the data were found suitable for illustrating the statistical analysis.

For 15 of the 68 analytes considered (namely 13 fatty acids, furfural and sodium) all results (or, in one case, all but one) were below a given limit value. Without further knowledge about the nature of this limit value we simply refer to it as the limit of reporting. As there was no variation in these results which could be used for a comparative evaluation, they were omitted from the further statistical analysis. Seven results in the remaining set of 53 analytes were reported as less than a given limit value (non-detects): six results for 16:1 palmitoleic acid and one result for phytic acid. The problem seemed minor, and, although more advanced statistical methods exist to incorporate such results in modelling, here the non-detects were simply set to half the limit of reporting. Outliers were identified by visual inspection of graphs showing the log-transformed results for each of the three groups (GMO, counterpart, reference). Outliers were identified for four analytes (see [11] for details), and also the seven non-detects set to half the limit of reporting were outlying. Outliers were omitted from the further statistical analysis.

A small additional simulation study was performed to investigate whether the observed number of significant differences between GMO and counterpart (23) is large under the null hypothesis that variation between genotypes can be described by a normal distribution with variance *V_g _*on the logarithmic scale. Here we take for *V_g _*the quantifications as obtained with the mixed model (Table 2). Under this null hypothesis and ignoring further estimation error, differences *d *between any two varieties would have a normal distribution with variance 2*V_g_*. In 1000 iterations random values for *d *were sampled from this distribution for all analytes, and a two-sided *t *test at the 95% confidence level was performed assuming that the *sed_GC;1 _*and the corresponding degrees of freedom estimate from the actual experiment were appropriate characterisations of residual error. Over the 1000 iterations the average number of significant test results was 36 (approximate 95% confidence interval (30, 42)). Therefore, under a null hypothesis describing equivalence between all the varieties, the observed number of significant differences between GMO and counterpart (23) is relatively small.

Differences between GMO and counterpart may not be constant over sites. This was investigated by fitting additional fixed terms ref_aside.site and ref_aside.genotypegroup.site in mixed model 1, and performing a Wald test to obtain a *p *value for the significance of the latter term. For 8 analytes the genotype by environment (GxE) interaction was significant (p < 0.05), and geometric means per site (not shown) may then help a further interpretation of the results.

## Availability and requirements

Programs implementing the proposed approach in the GenStat and SAS statistical packages for the specific example case data of this paper are available at http://www.efsa.europa.eu/en/scdocs/doc/1250ax2.pdf. More user-friendly and generally applicable software is under development and will be made available on the EFSA website later.

## Authors' contributions

HV developed the statistical model, performed the statistical analyses and drafted the manuscript. JNP participated in the statistical model development and the description of results. BA participated in the coding of the model in SAS. CP provided the regulatory background and was responsible for describing the position of EFSA. All authors contributed to drafts, and read and approved the final manuscript.
